# A case of endometriotic cyst enlargement during pregnancy owing to desmoplasia and rupture at 36 weeks of gestation

**DOI:** 10.1016/j.radcr.2022.10.053

**Published:** 2022-11-24

**Authors:** Shohei Tanabe, Sachiyo Sugino, Kotaro Ichida, Kiyoshi Niiya, Syuji Morishima

**Affiliations:** Kobe City Medical Center, West Hospital, 3-5-15-1001 Morigo-cho, Nada-ku, Kobe-shi, Hyogo, Japan

**Keywords:** Endometriotic cyst, Desmoplasia, Pregnancy, Ovarian cancer

## Abstract

Decidualized ovarian endometrioma is a rare phenomenon that occurs during pregnancy. A 43-year-old pregnant woman with an endometriotic cyst increased owing to desmoplasia presented to us urgently with abdominal pain and was performed a cesarean section at 36 weeks and 1 day of pregnancy. The left ovarian cyst was noted to be partially ruptured, and the pathological diagnosis was an endometriotic cyst with desmoplasia. Endometriotic cysts may enlarge during pregnancy owing to desmoplasia and rupture in the last trimester of pregnancy, causing acute abdomen.

## Introduction

Decidualized ovarian endometrioma is a rare phenomenon that occurs during pregnancy and must be differentiated from ovarian cancer [1]. We report the case of a decidualized ovarian endometrioma that became enlarged at 16 weeks of gestation, and after initial imaging and conservative follow-up it ruptured in the 36th week, resulting in acute abdomen. The patient provided written informed consent for publication of this report.

## Case report

**Fig. 1:**
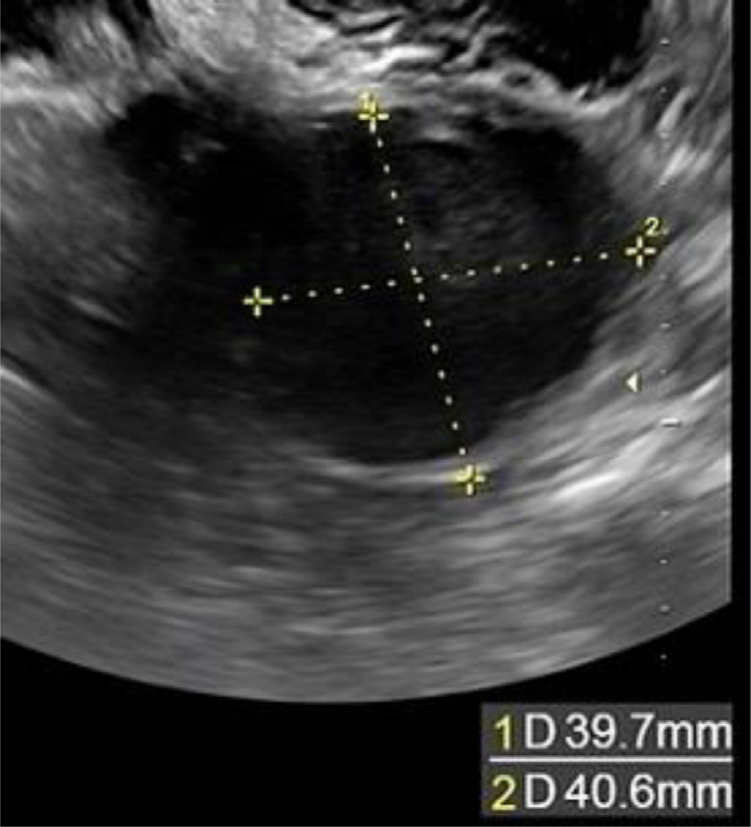
Ultrasound imaging before pregnancy. A 40-mm ovarian endometriotic cyst was noted.

**Fig. 2:**
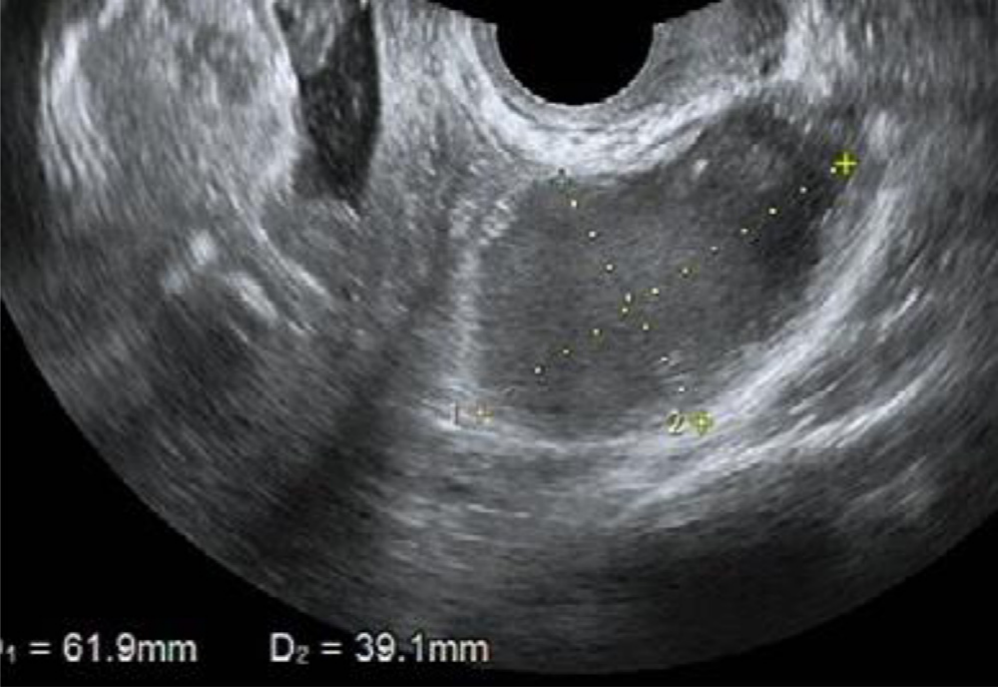
Ultrasound imaging at 16 weeks of pregnancy. The cyst increased in size to 60 mm.

**Fig. 3:**
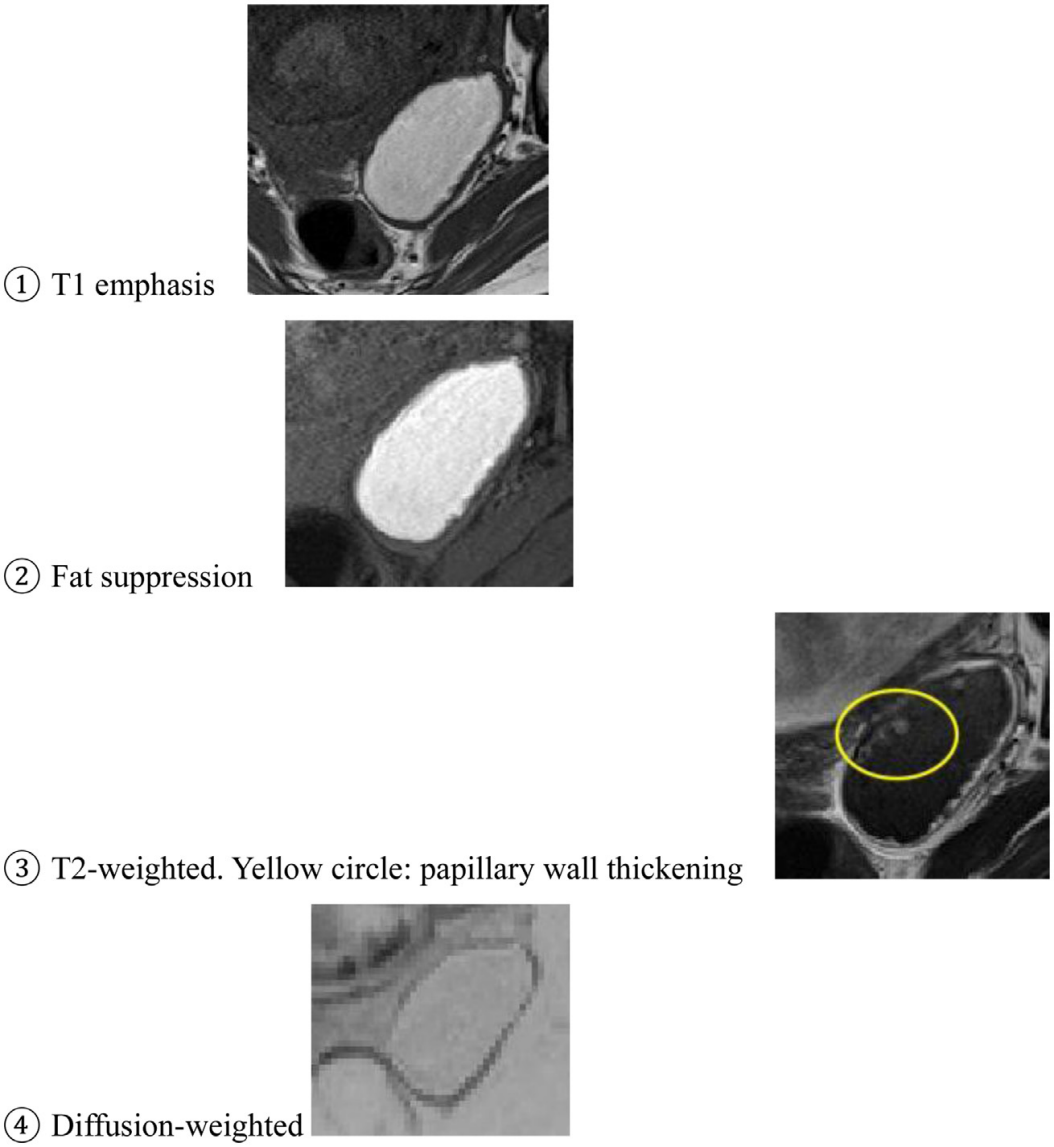
MRI imaging at 16 weeks of pregnancy. (A) T1-weighted image; (B) T1-weighted image with fat suppression; (C) T2-weighted image. Yellow circle: papillary wall thickening; (D) Diffusion-weighted image.

**Fig. 4:**
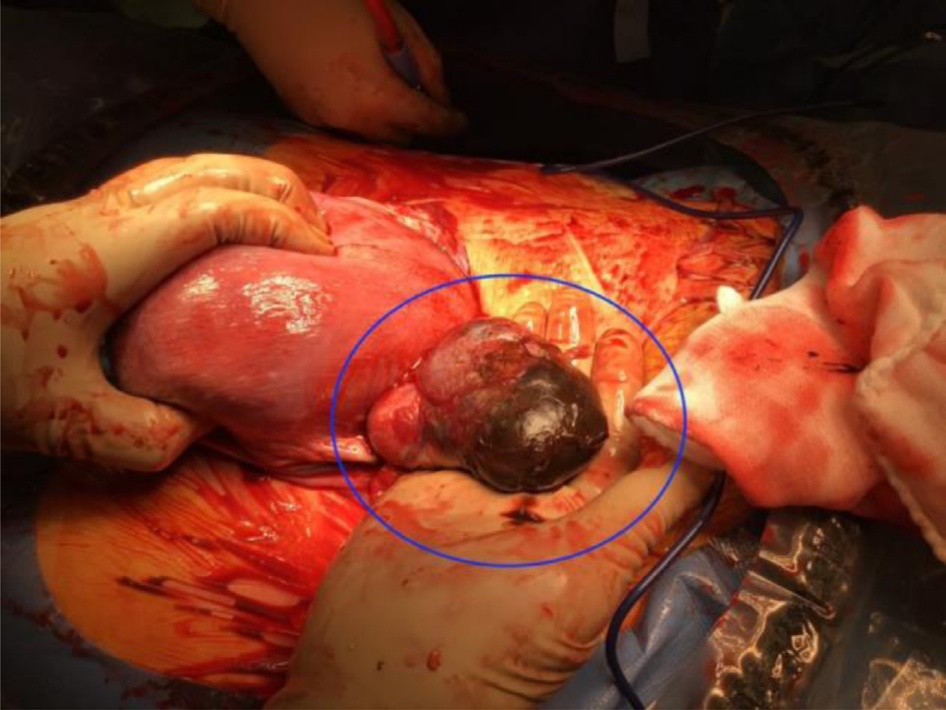
Surgical image. Blue circle: the left ovarian tumor.

**Fig. 5:**
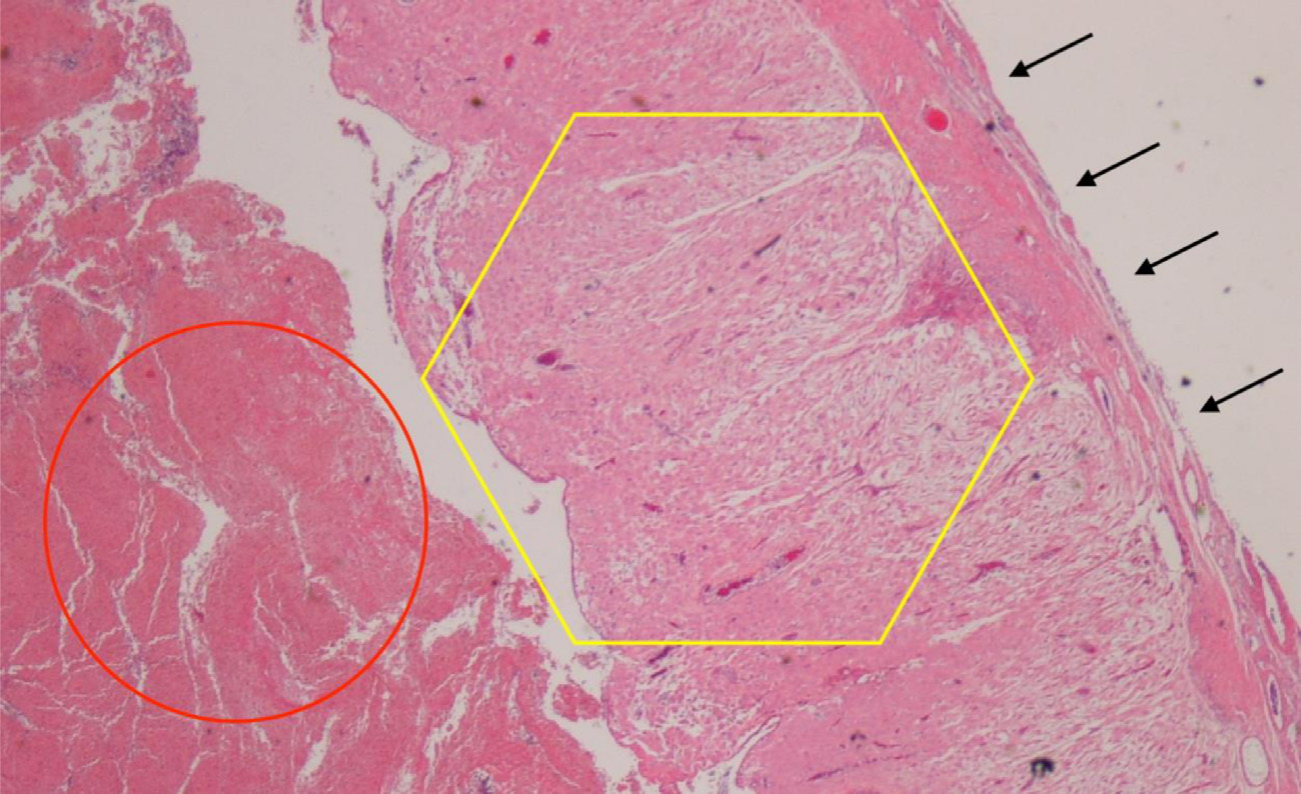
Histopathological image of the ovarian tumor. Hematoxylin-eosin staining, ×20 magnification. Red circle: internal hemorrhage; yellow hexagon: endometrial tissue; black arrows: ovarian wall.

A 43-year-old woman with 2 previous pregnancies and one lactation presented a 40-mm ovarian endometriotic cyst, noted before the following pregnancy (Fig. 1). At the 16-week pregnancy checkup, a transvaginal ultrasound showed that the endometrioid cyst had increased to 60 mm in size (Fig. 2), and an MRI revealed that the lesion was a 70-mm tumor, hyperintense on T1-weighted images and fat suppression and hypointense on T2-weighted images. Internally, the tumor showed some papillary wall thickening and no hyperintensity on diffusion-weighted MRI, suggesting desmoplasia of an endometrioid cyst (Fig. 3). The patient was followed conservatively without surgery during the remainder of the pregnancy. At 36 weeks and 0 days of pregnancy, the patient was urgently admitted due to sudden abdominal pain. Premature placental abruption was suspected; however, the cardiotocography findings were reassuring. However, intravenous acetaminophen was insufficient to resolve the symptoms, and a cesarean section was performed the following day. When the abdomen was opened, a small amount of brown ascites was found, and the left ovarian tumor was noted to be partially collapsed (Fig. 4). Enucleation of the tumor was performed in addition to the cesarean section. The postoperative pathology confirmed the diagnosis of an endometriotic cyst with desmoplasia (Fig. 5). The 1-month postoperative checkup showed no alterations in the ovary. The patient is currently under outpatient observation and plans another pregnancy after resumption of menstruation.

## Discussion

A decidualized ovarian endometrioma should be carefully distinguished from a malignant transformation, as it may show increased size and papillary projections on ultrasound imaging. MRI is useful to determine whether a malignant transformation occurred in endometriosis. The presence of a nodule with contrast effect is a finding suspicious for malignant transformation [1]. Because of her pregnancy, we could not use contrast media. Therefore, we concluded that malignancy was unlikely based on simple MRI images alone.

A previous review reported that a decidualized ovarian endometrioma diagnosed with MRI rarely causes complications that require surgery during pregnancy [2]. On the other hand, ovarian tumors >6 cm are at risk of rupture in gestation, and surgical resection is recommended [3]. Consequently, when a decidualized ovarian endometrioma ≥6 cm occurs during pregnancy, it is unclear whether the most appropriate strategy would be to follow up conservatively or perform surgery. A previous case report describes a patient treated with laparoscopic surgery at 25 weeks of gestation [4]. Laparoscopic surgery in pregnant women has currently become a standard procedure; therefore, this option may be considered more frequently than in the days when laparotomy was the only treatment available.

In conclusion, this case shows that endometriotic cysts may become desmoplastic and enlarged during pregnancy, requiring differentiation from malignancy. Endometriotic cysts increasing in size may also rupture in the last trimester of pregnancy and cause acute abdomen.

## Patient consent

Informed consent has been obtained from all individuals included in this study.

## References

[1] Takeuchi M, Matsuzaki K, Uehara H, Nishitani H. (2006). Malignant transformation of pelvic endometriosis: MR imaging findings and pathologic correlation. Radiographics.

[2] Frühauf F, Fanta M, Burgetová A, Fisherová D. (2019). Endometriosis in pregnancy—diagnostics and management. Ceska Gynekol.

[3] Ye P, Zhao N, Shu J, Ye P, Zhao N, Shu J (2019). Laparoscopy versus open surgery for adnexal masses in pregnancy: a meta-analytic review. Arch Gynecol Obstet.

[4] Yin M, Wang T, Li S, Zhang X, Yang J. (2022). Decidualized ovarian endometrioma mimicking malignancy in pregnancy: a case report and literature review. J Ovarian Res.

